# Feasibility of Ultra-Short-Term Analysis of Heart Rate and Systolic Arterial Pressure Variability at Rest and during Stress via Time-Domain and Entropy-Based Measures

**DOI:** 10.3390/s22239149

**Published:** 2022-11-25

**Authors:** Gabriele Volpes, Chiara Barà, Alessandro Busacca, Salvatore Stivala, Michal Javorka, Luca Faes, Riccardo Pernice

**Affiliations:** 1 Department of Engineering, University of Palermo, Viale delle Scienze, Building 9, 90128 Palermo, Italy; 2 Department of Physiology, Jessenius Faculty of Medicine, Comenius University, 036 01 Martin, Slovakia

**Keywords:** Heart Rate Variability (HRV), Short-Term (ST) cardiovascular variability, Ultra-Short-Term (UST) HRV, electrocardiography (ECG), Systolic Arterial Pressure (SAP), entropy, conditional entropy, complexity, time-series analysis

## Abstract

Heart Rate Variability (HRV) and Blood Pressure Variability (BPV) are widely employed tools for characterizing the complex behavior of cardiovascular dynamics. Usually, HRV and BPV analyses are carried out through short-term (ST) measurements, which exploit ~five-minute-long recordings. Recent research efforts are focused on reducing the time series length, assessing whether and to what extent Ultra-Short-Term (UST) analysis is capable of extracting information about cardiovascular variability from very short recordings. In this work, we compare ST and UST measures computed on electrocardiographic R-R intervals and systolic arterial pressure time series obtained at rest and during both postural and mental stress. Standard time–domain indices are computed, together with entropy-based measures able to assess the regularity and complexity of cardiovascular dynamics, on time series lasting down to 60 samples, employing either a faster linear parametric estimator or a more reliable but time-consuming model-free method based on nearest neighbor estimates. Our results are evidence that shorter time series down to 120 samples still exhibit an acceptable agreement with the ST reference and can also be exploited to discriminate between stress and rest. Moreover, despite neglecting nonlinearities inherent to short-term cardiovascular dynamics, the faster linear estimator is still capable of detecting differences among the conditions, thus resulting in its suitability to be implemented on wearable devices.

## 1. Introduction

In recent years, increased interest is being devoted to the study of the complex regulatory mechanisms of the human organism, in order to improve the level of knowledge of biological functions and the early detection of pathologies [1,2,3]. In particular, a large amount of information can be obtained by studying the cardiovascular system, both alone and through its interactions with other physiological systems, by analyzing several parameters, such as blood pressure, level of oxygenated hemoglobin, heart rate and heart rate variability (HRV) [4,5,6]. The latter represents the beat-to-beat variation of the duration of the cardiac cycle and allows information to be obtained not only about the cardiovascular function, but also about the balance between the activities of the sympathetic nervous system (SNS) and the parasympathetic nervous system (PNS), thus allowing a thorough understanding of the complex neuro-autonomic regulation. This is important, because the usually antagonistic actions of the SNS and the PNS vary in response to psycho-physical stress situations that can occur in different contexts during the daily life of both healthy and pathological individuals [7,8]. Similarly, the study of other cardiovascular parameters, such as systolic (SAP) and diastolic (DAP) arterial pressure variability, enables investigation into the multiple feedback (e.g., baroreflex control) and autoregulatory mechanisms (e.g., vascular myogenic autoregulation) that come into play in the complex cardiovascular regulation [9,10,11,12,13,14].

HRV is usually studied through the monitoring of electrocardiographic (ECG) recordings, extracting the time series of R–R intervals (i.e., the time periods between successive heartbeats) [6]. In clinical settings, the use of 24 h recordings, also referred to as long-term (LT) analysis, is considered the “gold standard” for the investigation of cardiovascular control mechanisms, since such timeframe allows a better description of the physiological processes, taking into account slower temporal fluctuations (e.g., the circadian rhythms) and the response of the organism to a wider range of external stimuli [6,11]. On the other hand, short-term (ST) measurements are typically based on 5 min recordings and have been more extensively employed for practical purposes, especially for assessing the balance between SNS and PNS activities, given that fluctuations mediated by autonomic nervous system (ANS), reflecting respiratory, baroreflex and vascular tone regulatory mechanisms overlap to generate short-term dynamics [6,9,10,11,15]. Similarly, blood pressure variability (BPV) can also be studied through short-term and long-term analyses; its variations have been shown to be the result of complex interactions between extrinsic environmental factors and intrinsic cardiovascular regulatory mechanisms, and have been associated with the risk of cardiovascular events and mortality [13,14,16].

Short-term HRV is commonly investigated through different time-, frequency- and information-theoretic domain indexes computed starting from ECG R-R interval time series. Specifically, time–domain indexes are used to quantify both average heart rhythm and the extent of beat-to-beat variability [6,17], while frequency–domain indexes extract information specific to various time scales of oscillations. Furthermore, more recently developed entropy-based measures permit the assessment of the regularity and complexity of cardiovascular dynamics [18,19,20,21,22]. ST HRV analysis has also been proven useful outside clinical settings, e.g., to monitor health and wellbeing at home and in everyday life scenarios using wearable smartwatches and smartbands [23,24,25,26].

With the widespread adoption of wearable biomedical devices, especially in domestic settings (e.g., smart-healthcare), research now focuses on whether, and to what extent, shorter recordings can be exploited for cardiovascular variability analysis, given their lower computational and memory resources, in order to quickly extract useful physiological indexes [23,24,27,28]. Several works have recently investigated on the so-called ultra-short-term (UST) HRV analysis, which exploits recordings shorter than 5 min, comparing the results with those obtained using the ST standard [29,30,31,32,33,34]. However, the choice of the time series length strongly influences the physiological indices derived from RR and blood pressure time series, in such a way that employing shorter recordings reduces the ability to resolve slower oscillations within the analyzed cardiovascular dynamics [17,35]. Generally, at least 2 min recordings are recommended to observe Low-Frequency (LF, range: 0.04–0.15 Hz) dynamics (related especially to SNS, but also to PNS activity) and at least 1 min to observe High-Frequency (HF, range: 0.15–0.4 Hz) dynamics (mainly related to parasympathetic activity fluctuations associated with respiration) [6,17]. Longer recordings, i.e., 24 h period LT analysis, further allow detection of lower frequency components, such as the very-low-frequency (VLF, 0.0033–0.04 Hz) and the ultra-low-frequency (ULF, <0.003 Hz). Therefore, the use of recordings shorter than 5 min may result in a loss of information related to slower dynamics if compared to ST analysis. The reliability of the cardiovascular parameters computed from UST recordings also depends on the acquisition protocol and on the dynamics of the response mechanisms to the task or stimulus [35]. In this sense, the use of validated and widely employed protocols used in HRV analysis could be envisaged, e.g., the passive head-up tilt test to evoke orthostatic stress [36,37].

The present work aims at evaluating the extent to which the loss of physiological information due to UST reduced time series length can be a good tradeoff for extracting physiological indices with lower real-time processing and storage costs, suitably for wearable devices [24,25,26,29,34,38]. Moreover, while several works have focused on ultra-short-term HRV [29,34,38,39], to the best of our knowledge there are no previous studies performing a UST blood pressure variability analysis. Herein, a comparison between UST and ST indices extracted in the time and information domains is performed on a dataset composed of systolic blood pressure (SAP) and interbeat interval (RR) time series acquired on a population of healthy subjects in rest and when undergoing orthostatic and mental stress. The analysis has been carried out by reducing the time series length from 300 (short-term, ≈5 min) to 60 samples (≈1 min), in steps of 60, to assess the loss of information at decreasing window length and to verify whether the shortest length is still able to discriminate the transition from rest to stress.

## 2. Materials and Methods

### 2.1. Experimental Protocol

Analyses were carried out on a historical dataset employed for assessing the effects of orthostatic and mental stress on cardiovascular dynamics. Data have been acquired from 61 healthy young volunteers (24 males, 37 females) aged 17.5 years ±2.4 years, normotensive, and with a normal body mass index (BMI = 19 ÷ 25 kg m^−2^) [40,41]. All participants signed a written informed consent form before taking part in the measurement protocol, also requiring a parental or legal guardian permission to participate in the study when the subject was a minor (i.e., less than 18 years of age). All procedures were approved by the Ethical Committee of the Jessenius Faculty of Medicine, Comenius University, Martin, Slovakia. Subjects were asked not to take substances influencing the autonomic nervous system and cardiovascular system activities [40,41].

Physiological signals recorded on the volunteers consisted of (i) electrocardiographic (ECG) signal acquired through a horizontal bipolar thoracic lead (CardioFax ECG-9620, NihonKohden, Tokyo, Japan), (ii) continuous arterial blood pressure (BP) recorded on the finger through the volume-clamp method (Finometer PRO, FMS, Amsterdam, The Netherlands). All signals were acquired synchronously with a sampling frequency of 1 kHz. 

**Figure 1:**
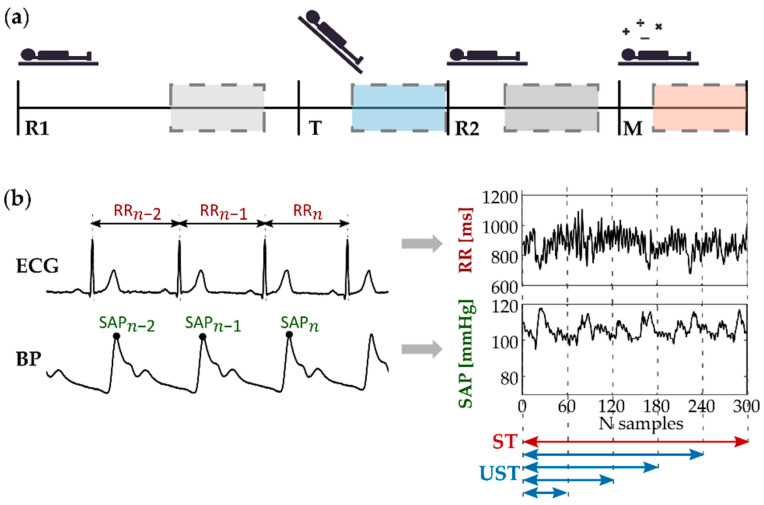
(**a**) Schematic illustration of the experimental protocol, including baseline resting (R1), orthostatic stress (T), second resting (R2) and mental stress (M). Dashed boxes indicate the windows taken into account with regard to short-term (ST, 300 points) analysis. (**b**) Representative RR and SAP time series, extracted respectively from ECG and BP recordings, which have been investigated through univariate analysis performed after ST (red arrow) and ultra-short-term (UST, 240 to 60 points, blue arrows) time window segmentation.

Subjects were positioned on a motorized tilt table and a restraining strap was placed at the thigh level to ensure the safety and stability of the subject during the movement of the tilt table. Signals were acquired during a measurement protocol consisting of the following four phases (schematically represented in Figure 1a):

- A resting condition (R1) with the subject laying in the supine position for 15 min, in order to stabilize the physiological signals on a baseline level;
- A head-up tilt (T) test aimed at evoking mild orthostatic stress by inclining the motorized table by 45 degrees for 8 min;
- Another resting condition (R2) with the subject laying in the supine position for 10 min, in order to restore the physiological parameters to their baseline values;
- A 6 min long mental arithmetic (M) task aimed to evoke cognitive load (i.e., mental stress), during which subjects were asked to mentally calculate the sum of three digits in the least possible time, indicating whether the result was an even or odd number.

During the whole measurement protocol, the subjects were asked to avoid any movement or speaking, to decrease artifacts occurrence and minimize the non-stationarities during recording of the signals.

### 2.2. Time Series Extraction

Starting from the ECG and BP signals acquired for each subject and condition, we extracted time series for carrying out the analyses described in the following subsections. Specifically, the RR interval time series was obtained by measuring the temporal distance between consecutive ECG QRS apexes, while the systolic arterial pressure (SAP) series was obtained as the sequence of the maximum values of the blood pressure signal measured within each RR interval. This procedure assured the same length for both RR and SAP time series, for each given subject and physiological condition.

The analyzed windows started 8 min after the beginning of the R1 phase, 3 min after the beginning of the T phase, 3 min after the beginning of the R2 phase, and 2 min after the beginning of the M phase (see schematization in Figure 1a). This choice was made in order to favor the stationarity of the time series, neglecting the transition effects due to the physiological changes elicited by the different phases during the measurement protocol [40,41]. Before performing the analyses, a visual inspection of the series was carried out to check for their stationarity.

Initially, time series of 300 heartbeats were extracted according to the standard of short-term analysis. The time series duration varied in the different conditions according to the heart rate, being on average ≈4.5 min during rest conditions, 3.5 min at T, and 4 min during M. Afterwards, in order to perform ultra-short-term (UST) analysis of cardiovascular parameters, shortened time series were obtained by reducing the series length each time of 60 samples down to a minimum of 60 heartbeats. The resulting UST time series were composed of 240, 180, 120, and 60 samples, selected starting from the beginning of the reference ST series, as schematized in Figure 1b.

### 2.3. Time-Domain Analysis

Time-domain analysis was performed on ST and UST RR and SAP time series computing the average (MEAN) and the standard deviation (STD) of the series values. With regard to RR time series, the standard deviation coincides with the widely used standard deviation of the interbeat intervals between normal sinus beats (SDNN) [6], given that no ectopic beats were detected. Moreover, for the RR time series, the root mean square of successive differences (RMSSD) was computed as follows to extract information about the beat-to-beat changes in heart rate mediated mostly by PNS [6,15,42]:

(1) $$RMSSD=\sqrt{\frac{1}{N-1}\overset{N-1}{\underset{n=1}{\sum }}{\left(x\left(n+1\right)-x\left(n\right)\right)}^{2}}$$

being x(n) the *n*-th RR samples and N the time series length.

### 2.4. Information Domain Analysis

For both the RR and SAP time series, information-theoretic analysis was performed to quantify the information carried by the physiological time series, as well as their complexity. The latter is typically quantified as the unpredictability of the present sample given its past samples, and thus has been associated to the regularity of the time series [22,43]. For this reason, in this work the static entropy (SE), dynamic entropy (DE), and conditional entropy (CE) measures were computed on either a short-term or ultra-short-term series using both a parametric and a model-free estimation.

Starting from a stationary stochastic process *X*, we can denote as $x=\left\{x_{1},\;x_{2},\ldots ,x_{N}\right\}$ the time series of length N, taken as a realization of the process *X*, as $X_{n}$ is the variable obtained by sampling the process *X* at the present time *n*, and $X_{n}^{m}=\left[X_{n-1},\;\ldots ,\;X_{n-m}\right]$ the variable describing the collection of the past *m* states. Using such notation, the static entropy quantifies the “static” information contained in the current state of the process *X*, without considering its temporal dynamics, and can be defined as [44]:

(2) $$SE=H\left(X_{n}\right)=-E\left[\log p\left(x_{n}\right)\right]$$

where E[·] is the expectation operator and p(·) the probability density, while H(·) denotes the entropy. The dynamic entropy (DE) instead represents the “joint” entropy of the present and past variables comprising the process; therefore, it provides the amount of information provided by the current sample of the series and by its past samples as well, thus providing “dynamic” information on the entire process. This can be defined as [45]:

(3) $$DE=H\left(X_{n},X_{n}^{m}\right)=-E[\log p\left(x_{n},\;x_{n-1},\;\ldots ,\;x_{n-m}\right)],$$

where H(·,·) is the joint entropy of two random variables. Then, the conditional entropy (CE) quantifies the average uncertainty that remains about the present state of the process when its past states are known (i.e., the new information contained in the current sample that cannot be inferred from the past history), and is defined as [44]:

(4) $$CE=H\left(X_{n}|X_{n}^{m}\right)=H\left(X_{n},\;X_{n}^{m}\right)-H\left(X_{n}^{m}\right)=-E\left[\log p(x_{n}|x_{n-1},\;x_{n-2},\;\ldots ,x_{n-m})\right]$$

where H(·|·) denotes conditional entropy operator.

In this work, the computation of SE, DE, and CE indices was carried out through two different estimation approaches, in order to identify which method allows the best trade-off between computational costs and ability to discriminate among physiological changes (i.e., rest versus stress). 

The first estimation method (hereinafter referred as *lin*) consists of a linear parametric approach based on the assumption that the observed process *X* is a stationary Gaussian process [44], which is a reasonable assumption given that many physiological data tend to follow a Gaussian distribution. Under this assumption, the above-mentioned entropies measures can be computed, after describing the dynamics of the process *X* with a linear regression model, from the covariance matrices of the variables sampling the process. In particular, the present and past variables of the process are related with the autoregressive (AR) model $X_{n}=A\cdot X_{n}^{m}+U_{n}$, where A is a vector of m regression coefficients and U is a white noise process modeling the prediction error. AR model identification has been performed via ordinary least squares method [46] to obtain estimations of regression parameters and prediction error variance, thus estimating the variance and the covariance matrices of the process. Then, denoting as ${\hat{\sigma }}_{X}^{2}$ the variance of the process, as ${\hat{\Sigma }}_{X_{n}X_{n}^{m}}$ the covariance matrix of the present and past states of *X*, and as ${\hat{\sigma }}_{U}^{2}$ the prediction error variance, the above defined entropy measures can be computed as [45]:

(5) $$SE_{lin}=\frac{1}{2}\ln\left(2\pi e{\hat{\sigma }}_{X}^{2}\right)$$

(6) $$DE_{lin}=\frac{1}{2}\ln\left({\left(2\pi e\right)}^{m+1}\left|{\hat{\Sigma }}_{X_{n}X_{n}^{m}}\right|\right)$$

(7) $$CE_{lin}=\frac{1}{2}\ln\left(2\pi e{\hat{\sigma }}_{U}^{2}\right)$$

where *e* is the Euler’s number.

The second estimation method (hereinafter referred as *knn*) is a model-free approach based on nearest neighbor metrics, which exploits the intuitive notion that the local probability density around a given data point is inversely related to the distance between the point and its neighbors. Using this approach, estimates of SE, DE and CE of the process *X* can be respectively computed through the following expressions [44,45]:

(8) $$SE_{knn}=\psi \left(N\right)+\langle \log\epsilon_{n,k}-\psi \left(N_{X_{n}}+1\right)\rangle$$

(9) $$DE_{knn}=-\psi \left(k\right)+\psi \left(N\right)+\left(m+1\right)\langle \log\epsilon_{n,k}\rangle$$

(10) $$CE_{knn}=-\psi \left(k\right)+\langle \log\epsilon_{n,k}+\psi \left(N_{X_{n}^{m}}+1\right)\rangle$$

where ψ(·) is the digamma function, *k* is the number of neighbors chosen for the analysis, $\epsilon_{n,k}$ represents twice the distance between the *n*-th realization of ($X_{n},X_{n}^{m}$) and its *k*-th nearest neighbor, $N_{X_{n}}$ and $N_{X_{n}^{m}}$, respectively, represent the number of points with a distance from $x_{n}$ and $x_{n}^{m}$ smaller than $\frac{\epsilon_{n,k}}{2}$ and ⟨·⟩ is the average operator; the average is taken over all the N−m realizations of the patterns ($X_{n},X_{n}^{m}$) that can be extracted from a series of length N. Here, estimation of the SE and CE in (8) and (10) has been performed, exploiting the distance projection method for bias compensation described in [44,45].

The ECG and SBP signals were pre-processed and analyzed using MATLAB R2021b (The MathWorks, Inc., Natick, MA, USA). All RR and SAP time series were normalized to zero mean before computing the three entropy measures. For computing DE and CE, the time series were also normalized to unit variance; this was not carried out for static entropy, whose calculation requires the knowledge of the information of the variance of the time series [45]. Information–domain indices were computed using the online available MATLAB ITS Toolbox (see Data availability statement section).

The number of neighbors chosen for the model-free estimator was *k* = 10, while the number of past components considered for the time series past histories was set equal to 2. Similarly, the order of the autoregressive model defined using standard least-squares regression for the *lin* approach was set to *m* = 2. 

In order to quantify the computational resources required to compute these indices, the time spent for calculating linear and model-free estimations of DE, CE and SE was measured for both RR and SAP time series in the four considered phases at varying time series lengths using the built-in MATLAB function.

### 2.5. Statistical Analysis

The statistical analyses have been carried out on distributions of time–domain and entropy measures obtained in the four phases (R1, T, R2, M) on both RR and SAP time series. Given that the normality of distributions for the analyzed indexes was verified according to the Kolmogorov–Smirnov test, the parametric Student’s *t*-test was used to perform the pairwise comparisons, with a significance threshold set to p<0.05. Specifically, the statistical tests were carried out to compare (i) orthostatic and mental stress conditions with resting states (i.e., T vs. R1 and M vs. R2) and (ii) ultra-short-term and short-term distributions (i.e., UST vs. ST).

However, the mere use of statistical tests has often been considered not sufficient for assessing the feasibility of the use of HRV indices evaluated through different techniques in studies where statistical tests have been complemented by other approaches, e.g., correlation analysis, Bland–Altman plots, or effect size [15,34,47,48]. In our work, correlation analysis was carried out through the computation of the squared Pearson correlation coefficient $r^{2}$ [49] to evaluate the strength of the linear relationship between each UST and the ST reference distribution, in order to quantify to what extent their agreement decreases when reducing the time series length. According to Shaffer et al. [38,50], who selected a conservative criterion for the Pearson correlation coefficient (*r* ≥ 0.90), herein we set a threshold for the squared coefficient equal to $r^{2}=0.81$ to establish the presence of a strong agreement between indexes derived from UST and ST analysis. 

Moreover, for all the time and information domain indices and time series length, we assessed the difference between the distributions during stress and during rest by computing the “effect size”. Measures of effect size represent a widely employed and useful tool to describe the strength of the association between two distributions, providing a description of the size of observed effects possibly independent of misleading influences on sample size, and thus can complement statistical tests that instead assess significance [51]. Large but nonsignificant effect sizes may indeed suggest that other statistical tests with greater discriminatory power should be employed, while small but significant effects due to large sample sizes can be indicative of overvaluing the observed effect [51]. In this work, the effect size has been evaluated through the Cohen’s *d* measure as the difference between the means of the two distributions divided by the pooled standard deviation [52]:

(11) $$d=\frac{\mu_{1}-\mu_{2}}{\sqrt{\frac{\left(n_{1}-1\right)\sigma_{1}^{2}+\left(n_{2}-1\right)\sigma_{2}^{2}}{n_{1}+n_{2}-2}}}$$

where μ, σ, and n are the mean, the standard deviation, and the number of samples of the two distributions under comparison (i.e., the number of subjects), respectively. Generally, the effect size is deemed as small, medium, and large, if the absolute value of *d* is lower than 0.2, between 0.2 and 0.5, or higher than 0.8, respectively. The Cohen’s *d* has been computed between stress and rest conditions (i.e., T vs. R1 and M vs. R2) for each time series length, in order to quantify whether and to what extent the strength of the relationship between the two distributions changes if compared to the ST reference. 

## 3. Results

### 3.1. Time-Domain Analysis

**Figure 2:**
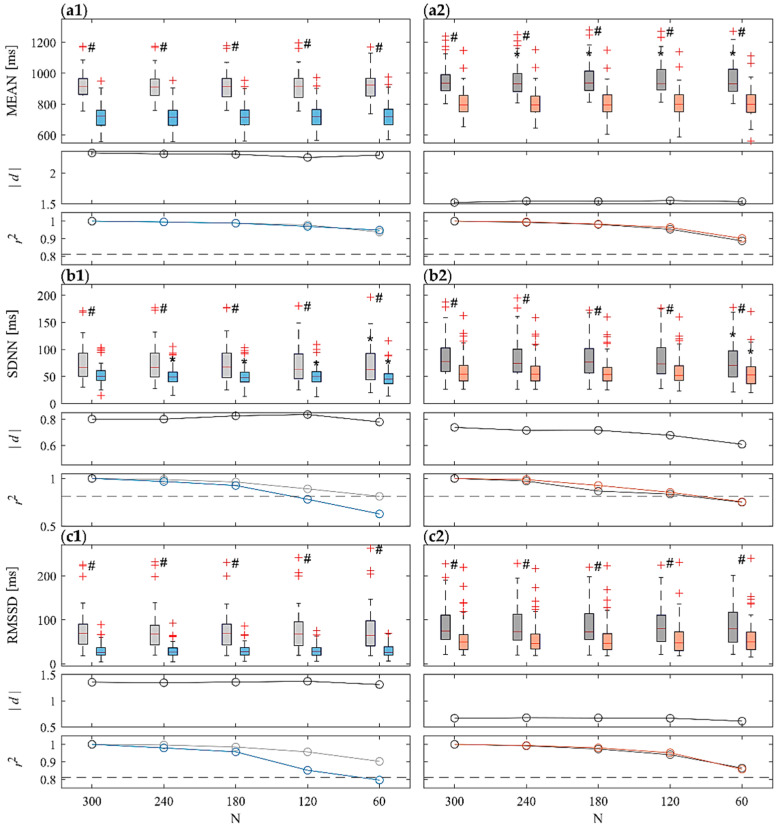
Boxplot distributions (top subplots) of time-domain indexes, i.e., (**a**) MEAN, (**b**) SDNN and (**c**) RMSSD calculated from RR time series during R1 (light gray) and T (light blue), (**.1** panels), and during R2 (dark gray) and M (orange) (**.2** panels) phases. Statistical tests: #, *p* < 0.05, T vs. R1 or M vs. R2; *, *p* < 0.05, ST vs. UST. Central subplots: Cohen’s *d* (in absolute value) evaluated between each stress condition and the previous rest phase (i.e., R1-T and R2-M) for all the considered time series lengths. Bottom subplots: squared Pearson correlation coefficients computed between a given UST distribution and the ST reference, with a threshold of $r^{2}=0.81$ (dotted gray line).

Figure 2 shows the comparison of ST (N = 300) and UST (N < 300) analyses performed for the time-domain indexes (MEAN, SDNN, RMSSD) computed over the RR time series across the 61 subjects in the four considered phases. For each panel, the top row subplot shows the boxplot distributions of the indices, the central one being the Cohen’s *d* measure (in absolute value), and the bottom one being the Pearson squared correlation coefficient. Results show a statistically significant decrease of all the three indexes during T vs. R1 and during M vs. R2 phases for the ST and for all the UST time window lengths taken into account. With regard to MEAN, statistically significant differences between UST and ST distributions are reported only for R2 already from N = 240 and for shorter time series (Figure 2(a2), top subplot). As regards SDNN, statistically significant differences have been detected comparing all the UST distributions to ST reference during head-up tilt (Figure 2(b1), top subplot). On the contrary, statistically significant differences have been reported only in the shortest UST distribution (N = 60) for both R2 and M (Figure 2(b2), top subplot). No statistically significant differences have been reported for RMSSD. Cohen’s *d* measures (central subplots in Figure 2) computed between stress and rest reported a medium-to-high effect size (|*d*| ≥ 0.7) for all the three indices, but lower for RMSSD during mental stress (|*d*| ≈ 0.5). Moreover, in all the cases the Cohen’s *d* showed higher values with regard to postural stress discrimination rather than mental stress. Furthermore, *d* remains almost constant at decreasing time series length, except for SDNN assessed during mental stress (Figure 2(b2)), in which it decreases with N. The squared Pearson correlation coefficient (bottom panels in Figure 2) computed between ST and UST distributions is very high and almost always above the threshold ($r^{2}>0.81$) for MEAN and RMSSD indices for all the time window lengths, except for RMSSD during T for *N* = 60. A considerable decrease in the correlation is reported for the SDNN index, going below threshold for T when N = 120 (Figure 2(b1), bottom panel), and for both R2 and M when N = 60 (Figure 2(b2), bottom panel). 

**Figure 3:**
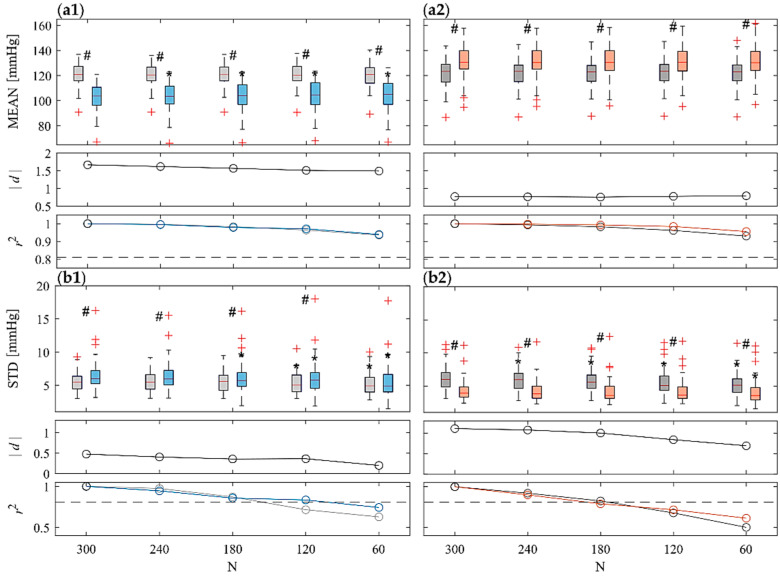
Boxplot distributions (top subplots) of time-domain indexes, i.e., (**a**) MEAN and (**b**) STD calculated from SAP time series during R1 (light gray) and T (light blue), (**.1** panels), and during R2 (dark gray) and M (orange) (**.2** panels) phases. Statistical tests: #, *p* < 0.05, R1 vs. T and R2 vs. M; *, *p* < 0.05, ST vs. UST. Statistical tests: #, *p* < 0.05, T vs. R1 or M vs. R2; *, *p* < 0.05, ST vs. UST. Central subplots: Cohen’s *d* (in absolute value) evaluated between each stress condition and the previous rest phase (i.e., R1-T and R2-M) for all the considered time series lengths. Bottom subplots: squared Pearson correlation coefficients computed between a given UST distribution and the ST reference, with a threshold of $r^{2}=0.81$ (dotted gray line).

Figure 3 shows the comparison of ST (N = 300) and UST (N < 300) analyses with regard to the time-domain indexes (MEAN, STD) of SAP time series across the 61 subjects in the four considered phases. With regard to ST, MEAN decreases significantly during T if compared to R1 (Figure 3(a1), top subplot), while it increases significantly during M if compared to R2 (Figure 3(a2), top subplot); opposite trends are reported with regard to STD (Figure 3(b1,b2), top subplots). For both indexes, results show statistically significant differences for T vs. R1 and for M vs. R2 phases for all the UST time window lengths taken into account, except for T vs. R1 with regard to the shortest series length (N = 60) only for STD (Figure 3(b1), top subplot). With regard to MEAN, statistically significant differences have been reported comparing UST vs. ST distributions during head-up tilt condition for N = 240 and shorter (Figure 3(a1), top subplot); on the other hand, no statistically significant differences have been reported with regard to R1 and to both M and R2 conditions (Figure 3(a2), top subplot)). As regards STD, statistically significant differences have been reported comparing UST vs. ST distributions for N ≤ 120 and N ≤ 180, respectively, for R1 and T (Figure 3(b1), top subplot)), for N ≤ 240 for R2, and for just N = 60 for M (Figure 3(b2), top subplot)). Cohen’s *d* values evidence a high effect size (*|d*| > 0.8), except for STD index during R1-T transition (Figure 3(b1), central subplot)) in which there is a medium-low effect size (|*d*| ≈ 0.5). An overall decrease in effect size is observed with the sample size *N*, especially for STD. The correlation analysis between UST and ST distributions reported a high squared correlation coefficient ($r^{2}>0.81$) with regards to MEAN distributions (Figure 3(a1,a2), bottom subplots). On the other hand, with regards to STD, the correlation coefficient strongly decreases when shortening *N*, going below the threshold for N = 120 for R1, R2 and M and for N = 60 for T (Figure 3(b1,b2), bottom subplots)). 

### 3.2. Information Domain Analyses

**Figure 4:**
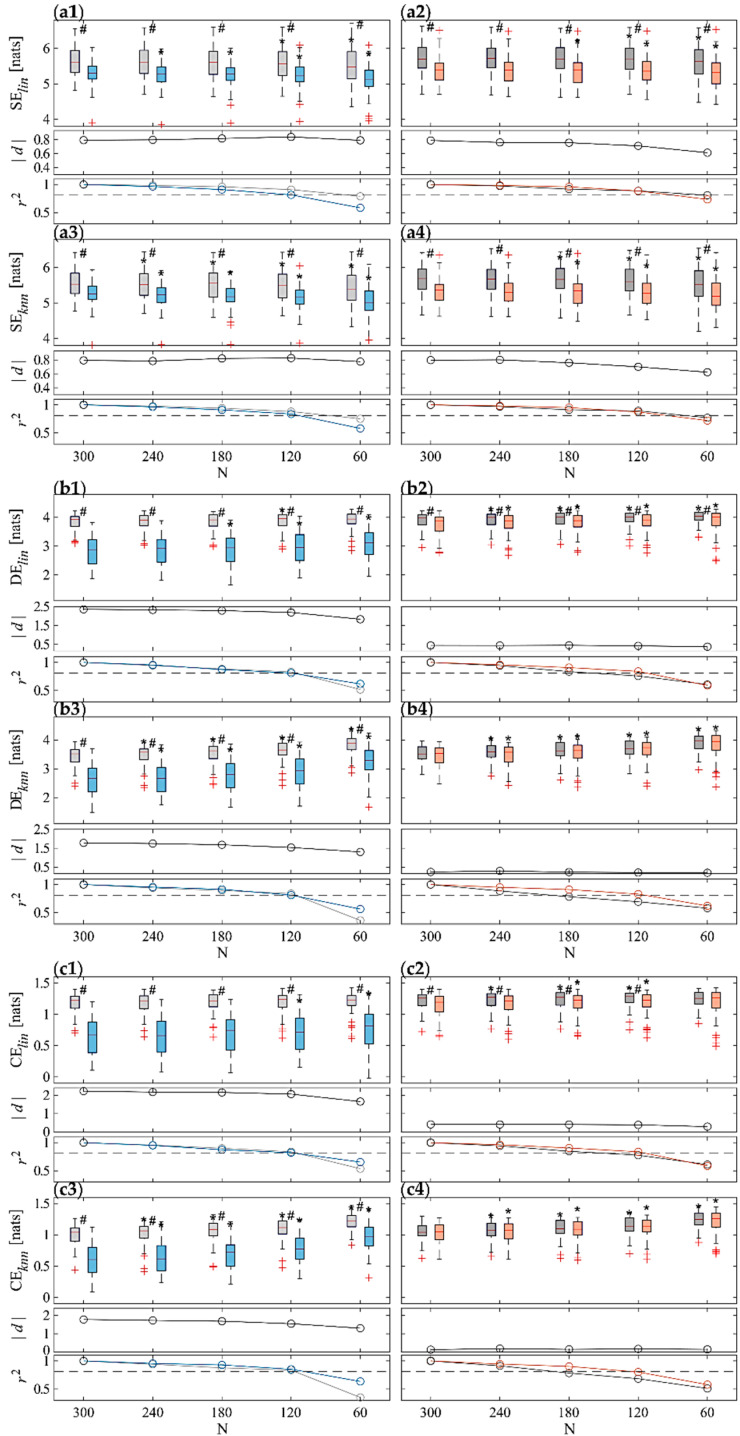
Results of information domain analysis on RR time series. Boxplot distributions (top subplots) of ST and UST indices of (**a**) SE, (**b**) DE and (**c**) CE calculated using both *lin* (.**1** and **.2**) and *knn* (**.3** and **.4**) estimators during R1 (light gray) and T (light blue) (**.1** and **.3**), and during R2 (dark gray) and T (orange) (**.2** and **.4**) phases. Statistical tests: #, *p* < 0.05, T vs. R1 or M vs. R2; *, *p* < 0.05 ST vs. UST. Central subplots: Cohen’s *d* (in absolute value) evaluated between each stress condition and the previous rest phase (i.e., R1-T and R2-M) for all the considered time series lengths. Bottom subplots: squared Pearson correlation coefficients computed between a given UST distribution and the ST reference, with a threshold of $r^{2}=0.81$ (dotted gray line).

**Figure 5:**
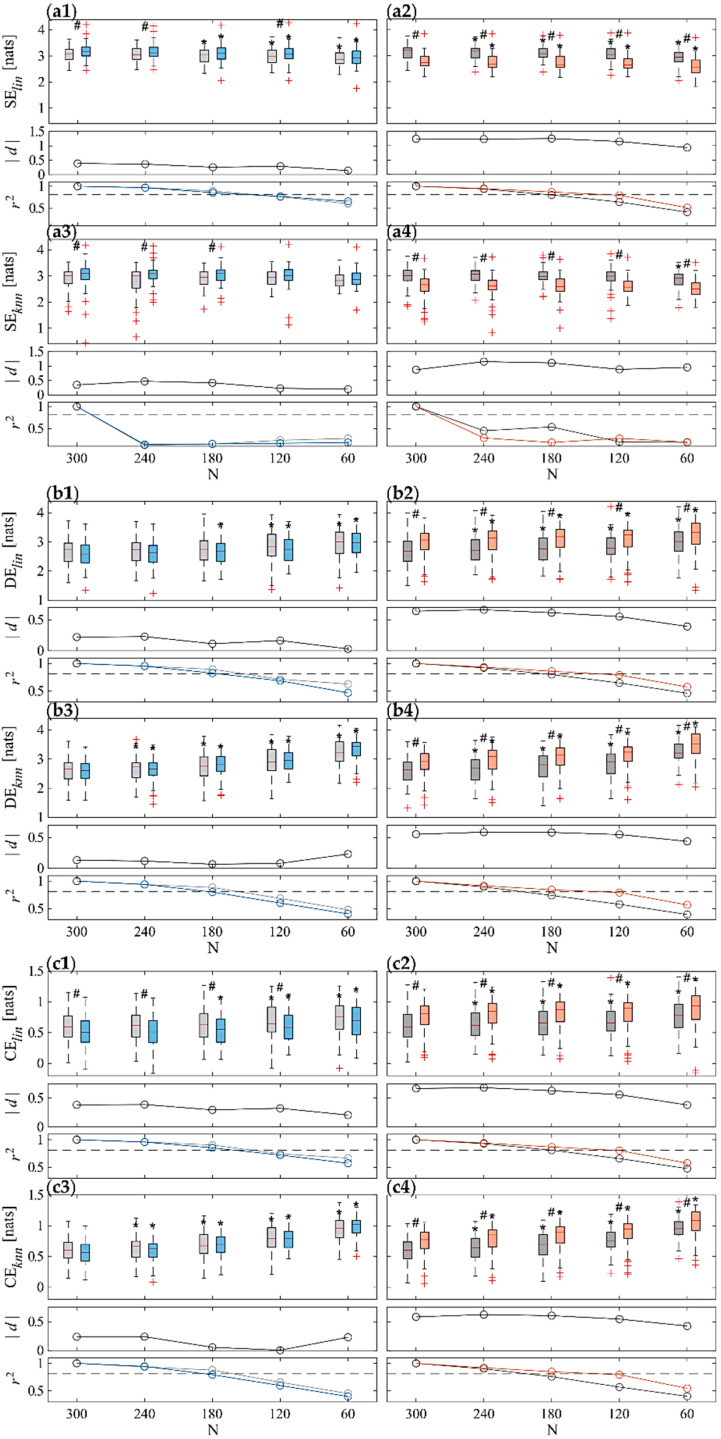
Results of information domain analysis on SAP time series. Boxplot distributions (top subplots) of ST and UST indices of (**a**) SE, (**b**) DE, and (**c**) CE calculated using both *lin* (.**1** and **.2**) and *knn* (**.3** and **.4**) estimators during R1 (light gray) and T (light blue) (.**1** and **.3**), and during R2 (dark gray) and T (orange) (**.2** and **.4**) phases. Statistical tests: #, *p* < 0.05, T vs. R1 or M vs. R2; *, *p* < 0.05 ST vs. UST. Central subplots: Cohen’s *d* (in absolute value) evaluated between each stress condition and the previous rest phase (i.e., R1-T and R2-M) for all the considered time series lengths. Bottom subplots: squared Pearson correlation coefficients computed between a given UST distribution and the ST reference, with a threshold of $r^{2}=0.81$ (dotted gray line).

Figure 4 and Figure 5 depict the results of the information domain analysis carried out by computing SE, DE, and CE indices through both *lin* and *knn* estimators for RR and SAP time series, respectively, across the 61 subjects for each of the four physiological conditions (R1, T, R2 and M). For each panel, the top row subplot shows the boxplot distributions of the indices, the central one the Cohen’s *d* measure (in absolute value), and the bottom one the Pearson squared correlation coefficient.

The results of the analyses carried out on RR time series are shown in Figure 4. For both orthostatic and mental arithmetic conditions, the shift from rest to stress highlights significant decrease of SE, DE and CE measures computed with both *lin* and *knn* estimators. This variation is most appreciable under the postural stress, in fact statistically significant variations are always reported comparing T vs. R1, for both ST and all the UST distributions and for both estimators. On the other hand, using the *lin* estimator statistically significant variations are reported comparing M vs. R2, for both ST and almost all the UST distributions for all measures (except w.r.t to CE for N = 60), but only for SE (Figure 4(a4)) using the *knn* approach. SE appears to decrease while CE and DE tend to increase when decreasing the time series length N. This result is more evident using the *knn* estimator, in fact statistical analysis carried out between UST and ST distributions highlighted significant differences starting from time series of length N = 240 for almost any measure computed through the model-free approach. For DE and CE computed through *lin* estimator, the statistically significant differences between UST and ST at T occur only for N **≤** 180 and N ≤ 120, respectively. The Cohen’s *d* values obtained for *lin* estimator are higher than for the *knn* one (see central subplots in each panel in Figure 4). High effect sizes are reported for CE and DE during T, but medium values instead during M; with regard to the SE index, a medium–high effect size is assessed for both physiological state changes and for both estimators. In any case, *d* appears almost constant at decreasing N (down to N = 120), while a more marked decrease is observed when going to N = 60. Finally, the squared Pearson correlation coefficient (see bottom subplots in each panel in Figure 4) decreases, shortening the time series length N, and still largely shows a high degree of correlation (above the threshold) down to N = 120.

As regards entropy measures computed on the SAP time series reported in Figure 5, both estimators (i.e., *lin* and *knn*) and analysis approaches (i.e., ST and UST) show an increase in SE (Figure 5(a1,a3), top subplots) as well as a decrease in DE (Figure 5(b1,b3), top subplots) and CE (Figure 5(c1,c3), top subplots) from R1 to T, and, conversely, a decrease in SE (Figure 5(a2,a4), top subplots) and an increase in DE (Figure 5(b2,b4), top subplots) and CE (Figure 5(c2,c4), top subplots) from R2 to M. Nevertheless, while differences between M and R2 distributions are always statistically significant, the comparison between T and R1 evidenced statistical significance only for *lin* estimation of CE using time series no shorter than 120 samples, and for SE obtained with both estimators and N ≤ 240. Regarding the comparison between ST and UST analyses, for both estimators and physiological state changes, CE and DE values increase as the time series length decreases, whereas the SE decreases more slowly. Statistical analysis highlighted significant differences already from the first window length (N = 240) for almost all the information indices obtained with both estimators during R2 and M, while overall this is true for T vs. R1 in any cases only for shorter time series (N < 180). The only exception is the *knn* estimation of SE, for which no significant differences are found between ST and UST analysis. The effect size assessed through Cohen’s *d* (Figure 5, central subplots in each panel) is always medium–low, except for SE index for M vs. R2, and overall decreases in absolute value when shortening the series length. The correlation analysis between UST and ST distributions (Figure 5, bottom subplots in each panel) shows that the squared Pearson’s correlation coefficient decreases when reducing time series length, still reporting values higher than the threshold ($r^{2}=0.81$) for almost all indices for N ≥ 180 (often even for N = 120), except for the SE estimated with the *knn* approach, for which $r^{2}$ severely drops already for N = 240 (Figure 5(a3,a4), bottom subplots). 

Finally, we report the results relevant to the computational times required for the calculation of the entropy-based measures, performed using both estimators. In order to compare ST and UST analysis times, we have selected N = 120 samples as UST time series length, since the previous results highlighted that this is the minimum length which overall guarantees a very good agreement between ST and UST distributions. The average computation time of all the entropy measures on 488 iterations (two time series in four different physiological conditions for 61 subjects) was 0.24 ms and 5.87 ms for *lin* and *knn* on ST 300-samples time series, respectively, and 0.17 ms and 1.90 ms on UST 120-samples time series. Such computational times were obtained on a computer equipped with an Intel Core i7-11700K CPU (3.60 GHz), 64 GB RAM, 512 GB SSD, Windows 11, MATLAB R2021b. The computational times are similar for RR and SAP series and do not vary as well with the protocol phase. Moreover, while computational times remain almost constant as time series length decreases with regard to *lin*, they strongly decrease at shortening N with regard to model-free estimation.

## 4. Discussion

The aim of this work was to evaluate the extent to which UST HRV and BPV measurements of less than 5 min duration can be used as a substitute for widely employed and validated short-term recordings. Employing standard time-domain and recently introduced entropy-based measures, we aim to assess whether and which UST metrics can replace ST indices to discriminate postural and mental stress states, while exploiting shorter recordings that are less prohibitive in terms of time cost, and computational power. In this sense, we also compared two different approaches to estimating entropy-based measures, i.e., a more reliable but also more computationally intense non-linear model-free method, and a faster but less general linear model-based approach. The rest of the discussion is organized as follows. Section 4.1 and Section 4.2 focus on physiological interpretation of results obtained through ST time-domain and information-theoretic domain metrics, respectively. Section 4.3 discusses UST results in terms of their agreement with standardized ST measures.

### 4.1. Time-Domain Analyses on ST Series

Time-domain HRV results (Figure 2) are in agreement with widely recognized findings in the literature which evidence an increased heart rate and a decrease of variability (SDNN) and RMSSD during stress conditions, in particular after head-up tilt [15,53,54]. In both stress conditions, but less markedly during mental stress, these trends are related to an enhanced sympathetic and reduced parasympathetic activity, resulting from an SNS activation and a PNS withdrawal which cause a shift in the sympathovagal balance [55,56,57]. In particular, the reduced parasympathetic contribution is evidenced by the decreased RMSSD (Figure 2(c1,c2)) which has been usually related to PNS activity [6,17]. Nevertheless, physiological mechanisms involved during orthostatic and arithmetical stress are different, as demonstrated by the different SAP MEAN and STD trends in these two stress conditions (cf. Figure 3(a2) vs. Figure 3(a1) and Figure 3(b2) vs. Figure 3(b1)). This is in agreement with previous studies highlighting the presence of a closed-loop regulatory mechanism between RR and SAP [10,58,59]. The decrease of the mean SAP together with the increase of its variability during postural stress (Figure 3(a1,b1)) have been related to the decreased venous return [60,61,62]. The resulting cardiac filling associated with SAP decrease leads to baroreflex activation and to vasoconstriction during postural stress, which in turn produce an increased heart rate [60,61,62,63]. The opposite trends reported for mental stress (cf. Figure 3(a2) vs. Figure 3(b2) can be related to cortical mechanisms eliciting vasomotor reactions and are reflected by SAP changes [54,64,65].

### 4.2. Information Domain Analyses on ST Series

The shift of the autonomic balance to the sympathetic branch caused by orthostatic and cognitive challenges produces a simplification of the cardiac dynamics, with reduced information contained in the RR time series (Figure 4(a1–a4)), which has been linked to the emergence of oscillations at the frequency of the Mayer waves [15,41]. The elicited stress conditions lead also to a decrease in complexity, and thus lower CE and DE values using both linear and non-linear estimators (Figure 4(b1–c4)). Physiologically, this indicates a regularizing effect on the cardiac dynamics produced by sympathetic activation and vagal withdrawal already demonstrated in several previous works also on the same dataset [15,22,43,66,67].

The entropy-based SAP analysis revealed opposite trends for T and M compared to the preceding resting condition (cf. Figure 5 right vs. left panels), confirming the different response to postural and mental stress. Mental challenge produced an SE decrease and increased complexity, while opposite trends have been observed for postural stress. These findings evidence that SAP dynamics are less affected by orthostatic stress than by cognitive load. Physiologically, this can be ascribed to the larger involvement of upper brain centers in controlling the vascular dynamics and resistance associated with sympathetic activation. A relatively complex pattern of vascular resistance changes results in an augmented SAP dynamical complexity, as demonstrated by previous works [41,43,64,67,68]. The trend towards lower SAP complexity values during tilt may be related to the synchronization of peripheral vascular activity due to sympathetic activation, contributing to regularizing the fluctuations of SAP [69].

Comparing the entropy measures obtained through the two estimators, we found lower values using *knn* than using the *lin* approach, especially with regard to RR and under rest and mental stress conditions. The reasons of such a difference are difficult to explain and may be related to several factors, ranging from local nonlinearities or nonstationarities to bias effects evident especially for the *knn* estimator and due to the difficulty of working on high-dimensional spaces [70]. Nevertheless, for the majority of measures both estimators exhibit concordant changes and are equally able to distinguish between rest and stress conditions. There are three exceptions, in which the *knn* is unable to detect differences, while *lin* does, i.e., CE for RR comparing M vs. R2 (Figure 4(c4) vs. Figure 4(c2)), DE for RR comparing M vs. R2 (cf. Figure 4(b4) vs. Figure 4(b2)), and CE for SAP comparing T vs. R1 (cf. Figure 5(c3) vs. Figure 5(c1)). The augmented discriminative capability of the linear estimator, even if possibly related to the presence of non-linear dynamics [71,72] which are not properly taken into account, may be a perspective used in practical applications for a more accurate and fast differentiation between rest and stress conditions [39,67]. This is also reinforced by the very low computational times required for the *lin* estimator to compute the entropy-based measures on 300-sample series length (ST standard), which is 24 times lower if compared to *knn*.

### 4.3. Ultra-Short-Term versus Short-Term Analysis

The main focus of this work was to assess whether using shorter heart rate and SAP time series allows us to obtain the same physiological information extracted with ST series, as discussed in the previous Section 4.1 and Section 4.2. Regarding the reliability of using UST RR time series to discriminate between stress and rest conditions, our time-domain results (Figure 2) demonstrate that, overall, it is possible to make use of 60-sample recordings to detect the presence of either postural or mental stress compared to a rest condition. This is true despite the fact that statistically significant differences between UST and ST series are detected in R2 with regard to MEAN, and in T with regard to SDNN even for 240-sample time series (Figure 2(a2,b1)). Therefore, our results suggest that, while UST analysis implies a significant deviation of the analyzed metrics from their ST level, such deviation does not significantly impair the capability to detect the response to stress even when working with shorter time series.

The above-discussed results are reinforced by correlation analysis, which reported squared Pearson correlation coefficient always above the adopted threshold for strong correlation, with the only exception being SDNN computed for N = 60. Analogous remarks can be made starting from Cohen’s *d* analysis between stress and rest conditions, with similar values for all time series lengths, with only a noticeable decrease for N = 60. Such results are in agreement with previous studies in the literature on RR series reporting a good agreement between UST and ST both under physical stress [30,73] and mental stress [29,74] conditions. However, the agreement decreases during the execution of a task or in the presence of a stressful event that carries dynamicity in the control mechanisms [33,48,75], similar to the trends reported in our results with regard to squared Pearson correlation coefficient. A number of studies applying UST analysis to physical stress conditions focus on the following recovery phase, showing that the dynamics are strongly influenced by the intensity of the task and the response time of SNS and PNS [30,76]; this may explain the statistically significant differences found in R2 with regard to MEAN, being R2 a post-postural stress rest. 

A quite common finding in previous works is that the SDNN index exhibits a lower agreement if computed through UST RR series [48,77], and this is confirmed by our results analyzing the trend of the correlation coefficient (Figure 2(b1,b2), bottom subplots). On the other hand, the agreement is higher with regard to RMSSD index (Figure 2(c1,c2), bottom subplots). This finding appears to be directly related to the definition of metrics, since whereas SDNN reflects RR total power, the RMSSD is instead related only to the fastest variations (i.e., vagally-mediated ones) observable even from shorter time series [17]. Although it is not possible to refer to previous studies, the results obtained with regard to SAP (Figure 3) can be discussed similarly to RR. In particular, results highlight the capability of using UST SAP time series to discriminate between stress and rest conditions down to N = 60 (with the exception of STD for postural stress), even if statistically significant differences are reported between UST and ST distributions for MEAN during T and for most conditions for STD. Similarly to RR, a very high squared Pearson correlation coefficient is reported between UST and ST distributions, decreasing with N and going below threshold for N ≤ 120 for STD. Likewise to RR, the agreement of the STD measure is lower for UST series, which is also confirmed by the lower effect size between the rest and stress conditions.

Our results confirmed the feasibility of employing UST series to carry out computation of regularity and complexity measures (Figure 4 and Figure 5), already previously reported for CE and Approximate Entropy [29,39,78]. Results of statistical tests evidence that, apart from a couple of exceptions, the significant differences between the stressful and the preceding rest conditions reported using 300-sample recordings are also retained for all the analyzed metrics (SE, DE, CE) with shorter series down to a 60-sample duration. However, the results of correlation analysis have evidenced that the agreement between UST and ST distributions is overall very good (i.e., above threshold) only for N ≥ 120 for RR and for N ≥ 180 for SAP, except for non-parametric SE that already exhibits a severe decrease of $r^{2}$ from 4 min length recordings. The results of RR analyses are in agreement with some previous studies employing other non-linear measures for the analysis of predictability (e.g., Shannon Entropy [29,33]), dynamics (e.g., Permutation Entropy [79]), and complexity (e.g., Approximate Entropy and Sample Entropy [29,33,79]), overall reporting that recordings of at least 2–3 min are necessary in order to have good consistency with respect to ST standard. Our results complement these findings, supporting the hypothesis that the variation in cardiovascular dynamics and the complexity produced by a physiological state change can be properly assessed using even shorter recordings (60 samples), but at the cost of a lower correlation with ST reference. Moreover, the lower correlation found for SAP in our analyses suggests that slightly longer recordings may be instead envisaged when performing UST blood pressure variability if compared to HRV.

The Cohen’s *d* analysis on entropy measures evaluated on RR evidenced a better discrimination of postural stress than mental stress with a higher effect size (Figure 4, central subplots), in contrast to what is evidenced instead with regard to SAP (Figure 5, central subplots). In any case, the effect size decreases when shortening the time window length, thus suggesting a lower discriminative capability between stress and rest states caused by the information loss about slower dynamics due to the shorter time series.

Finally, regarding the comparison of estimation methods for entropy-based measures, the same considerations made in Section 4.2 also hold for the UST analysis. For the majority of measures, both estimators are similarly capable of distinguishing between rest and stress conditions. There are the same three exceptions discussed with regard to ST series, in which the *knn* is unable to detect differences while *lin* can, i.e., CE for RR comparing M vs. R2 (Figure 4(c4) vs. Figure 4(c2)), DE for RR comparing M vs. R2 (Figure 4(b4) vs. Figure 4(b2)), and CE for SAP comparing T vs. R1 (Figure 5(c3) vs. Figure 5(c1)). This may be due again to the significant proportion of nonlinear dynamics also contributing to UST HRV and cardiovascular variability [41,72,80] that are detected by *knn* estimator but neglected by the model-based parametric approach. Also in this case, the increased discriminative capability of the linear estimator and its lower computational costs may be exploited for discrimination between rest and stress conditions [67]. Furthermore, our results demonstrate that using shorter time series also requires reduced computational costs for both estimators, with a decrease of ~1.7 times for *lin* and ~3.1 times for *knn* when shortening the time series length from 300 to 120 samples. Computing all the information indices exploiting time series of 120 samples through the parametric estimator is ~11 times less computationally expensive than using the model-free estimator. 

## 5. Conclusions

In this work, a comparison between ST and UST analysis has been carried out by computing physiological indices in time and information-theoretic domains on heart rate and blood pressure variability time series, during rest and both orthostatic and mental stress conditions, to assess to what extent UST analysis can represent a valid substitute for the ST standard, especially for stress detection. 

Our results showed that time-domain and entropy-based measures computed on RR and SAP series are able to discriminate between rest and stress even for very short time series length, down to N = 120 or even N = 60 samples in most cases. However, the drop in correlation below the set threshold reported for UST shortest windows (N ≤ 120) suggests caution in the use of very-short-time series segments, especially when analyzing SAP variability, opening a new issue that deserves further investigation in the future. 

Finally, the comparison between a more reliable but time-consuming model-free estimator and a linear model-based approach suggests that the latter can be suitably employed for detecting changes in physiological conditions, thanks to the considerable benefits in terms of reduction of computational costs at the expense of information loss about non-linearities in cardiovascular dynamics. 

The combined use of UST series and faster linear estimators for entropy-based measures can be beneficial for the integration of such metrics within wearable devices for a real-time monitoring of cardiovascular parameters. Moreover, the large number of features computed from data acquired by wearable devices could be employed in the future to develop accurate machine-learning-based classifiers for stress detection. A future extension of this work could focus on comparing UST indices in different classes of subjects, e.g., to evaluate differences due to sex, age, or body mass index. A further activity could be devoted to performing the same UST analyses on photoplethysmographic (PPG) signals, which can be directly acquired on wearable devices. Several studies agree on considering pulse rate variability assessed from PPG as a surrogate of heart rate variability [15,81,82], and recent studies have also investigated the use of PPG time series shorter than 300 samples as an alternative for the standard ST analysis [31,83]. Moreover, analyzing PPG signals also allows information to be extracted on blood pressure [84], and this would to achieve insight into both heart rate and blood pressure variability which, as seen from our results, often yield complementary results.

## Data Availability

The data presented in this study will be made available on request from the corresponding author. The software relevant to the estimation of information-domain measures is part of the ITS toolbox, which is freely available for download at www.lucafaes.net/its.html, accessed on 28 October 2022.

## References

[1] Chaddha A., Robinson E.A., Kline-Rogers E., Alexandris-Souphis T., Rubenfire M. (2016). Mental Health and Cardiovascular Disease. Am. J. Med..

[2] Huang C.-J., Webb H.E., Zourdos M.C., Acevedo E.O. (2013). Cardiovascular Reactivity, Stress, and Physical Activity. Front. Physiol..

[3] Doherty A.M., Gaughran F. (2014). The Interface of Physical and Mental Health. Soc. Psychiatry Psychiatr. Epidemiol..

[4] Freeman J.V., Dewey F.E., Hadley D.M., Myers J., Froelicher V.F. (2006). Autonomic Nervous System Interaction with the Cardiovascular System during Exercise. Prog. Cardiovasc. Dis..

[5] Monti A., Medigue C., Nedelcoux H., Escourrou P. (2002). Autonomic Control of the Cardiovascular System during Sleep in Normal Subjects. Eur. J. Appl. Physiol..

[6] Shaffer F., Ginsberg J.P. (2017). An Overview of Heart Rate Variability Metrics and Norms. Front. Public Health.

[7] Falcone C., Colonna A., Bozzini S., Matrone B., Guasti L., Paganini E.M., Falcone R., Pelissero G. (2014). Cardiovascular Risk Factors and Sympatho-Vagal Balance: Importance of Time-Domain Heart Rate Variability. J. Clin. Exp. Cardiol..

[8] Shaffer F., McCraty R., Zerr C.L. (2014). A Healthy Heart Is Not a Metronome: An Integrative Review of the Heart’s Anatomy and Heart Rate Variability. Front. Psychol..

[9] Malpas S.C. (2002). Neural Influences on Cardiovascular Variability: Possibilities and Pitfalls. Am. J. Physiol. Circ. Physiol..

[10] Schulz S., Adochiei F.-C., Edu I.-R., Schroeder R., Costin H., Bär K.-J., Voss A. (2013). Cardiovascular and Cardiorespiratory Coupling Analyses: A Review. Philos. Trans. R. Soc. A Math. Phys. Eng. Sci..

[11] Cohen M.A., Taylor J.A. (2002). Short-Term Cardiovascular Oscillations in Man: Measuring and Modelling the Physiologies. J. Physiol..

[12] Porta A., Baselli G., Cerutti S. (2006). Implicit and Explicit Model-Based Signal Processing for the Analysis of Short-Term Cardiovascular Interactions. Proc. IEEE.

[13] Parati G., Di Rienzo M., Ulian L., Santucciu C., Girard A., Elghozi J.-L., Mancia G. (1998). Clinical Relevance Blood Pressure Variability. J. Hypertens. Suppl. Off. J. Int. Soc. Hypertens..

[14] Mancia G., Parati G., Di Rienzo M., Zanchetti A., Zanchetti A., Mancia G. (1997). Blood Pressure Variability. Handbook of Hypertension, Vol 17: Pathophysiology of Hypertension.

[15] Pernice R., Javorka M., Krohova J., Czippelova B., Turianikova Z., Busacca A., Faes L. (2019). Comparison of Short-Term Heart Rate Variability Indexes Evaluated through Electrocardiographic and Continuous Blood Pressure Monitoring. Med. Biol. Eng. Comput..

[16] Parati G., Ochoa J.E., Lombardi C., Bilo G. (2013). Assessment and Management of Blood-Pressure Variability. Nat. Rev. Cardiol..

[17] Camm A.J., Malik M., Bigger J.T., Breithardt G., Cerutti S., Cohen R.J., Coumel P., Fallen E.L., Kennedy H.L., Kleiger R.E. (1996). Heart Rate Variability: Standards of Measurement, Physiological Interpretation and Clinical Use. Task Force of the European Society of Cardiology and the North American Society of Pacing and Electrophysiology. Circulation.

[18] Castiglioni P., Parati G., Faini A. (2019). Information-Domain Analysis of Cardiovascular Complexity: Night and Day Modulations of Entropy and the Effects of Hypertension. Entropy.

[19] Porta A., Castiglioni P., Bari V., Bassani T., Marchi A., Cividjian A., Quintin L., Di Rienzo M. (2012). K-Nearest-Neighbor Conditional Entropy Approach for the Assessment of the Short-Term Complexity of Cardiovascular Control. Physiol. Meas..

[20] Sassi R., Cerutti S., Lombardi F., Malik M., Huikuri H.V., Peng C.-K., Schmidt G., Yamamoto Y., Reviewers: D., Gorenek B. (2015). Advances in Heart Rate Variability Signal Analysis: Joint Position Statement by the e-Cardiology ESC Working Group and the European Heart Rhythm Association Co-Endorsed by the Asia Pacific Heart Rhythm Society. Ep Eur..

[21] Richman J.S., Moorman J.R. (2000). Physiological Time-Series Analysis Using Approximate Entropy and Sample Entropy. Am. J. Physiol. Circ. Physiol..

[22] Porta A., Guzzetti S., Furlan R., Gnecchi-Ruscone T., Montano N., Malliani A. (2006). Complexity and Nonlinearity in Short-Term Heart Period Variability: Comparison of Methods Based on Local Nonlinear Prediction. IEEE Trans. Biomed. Eng..

[23] Chen C.M., Anastasova S., Zhang K., Rosa B.G., Lo B.P.L., Assender H.E., Yang G.Z. (2020). Towards Wearable and Flexible Sensors and Circuits Integration for Stress Monitoring. IEEE J. Biomed. Health Inform..

[24] Dias D., Cunha J.P.S. (2018). Wearable Health Devices—Vital Sign Monitoring, Systems and Technologies. Sensors.

[25] Umair M., Chalabianloo N., Sas C., Ersoy C. (2021). HRV and Stress: A Mixed-Methods Approach for Comparison of Wearable Heart Rate Sensors for Biofeedback. IEEE Access.

[26] Hernando D., Roca S., Sancho J., Alesanco Á., Bailón R. (2018). Validation of the Apple Watch for Heart Rate Variability Measurements during Relax and Mental Stress in Healthy Subjects. Sensors.

[27] Kos A., Umek A. (2019). Wearable Sensor Devices for Prevention and Rehabilitation in Healthcare: Swimming Exercise with Real-Time Therapist Feedback. IEEE Internet Things J..

[28] Kakria P., Tripathi N., Kitipawong P. (2015). A Real-Time Health Monitoring System for Remote Cardiac Patients Using Smartphone and Wearable Sensors. Int. J. Telemed. Appl..

[29] Castaldo R., Montesinos L., Melillo P., James C., Pecchia L. (2019). Ultra-Short Term HRV Features as Surrogates of Short Term HRV: A Case Study on Mental Stress Detection in Real Life. BMC Med. Inform. Decis. Mak..

[30] Kim J.W., Seok H.S., Shin H. (2021). Is Ultra-Short-Term Heart Rate Variability Valid in Non-Static Conditions?. Front. Physiol..

[31] Finžgar M., Podržaj P. (2020). Feasibility of Assessing Ultra-Short-Term Pulse Rate Variability from Video Recordings. PeerJ.

[32] Holmes C.J., Fedewa M.V., Winchester L.J., MacDonald H.V., Wind S.A., Esco M.R. (2020). Validity of Smartphone Heart Rate Variability Pre-and Post-Resistance Exercise. Sensors.

[33] Shaffer F., Shearman S., Meehan Z.M. (2016). The Promise of Ultra-Short-Term (UST) Heart Rate Variability Measurements. Biofeedback.

[34] Pecchia L., Castaldo R., Montesinos L., Melillo P. (2018). Are Ultra-short Heart Rate Variability Features Good Surrogates of Short-term Ones? State-of-the-art Review and Recommendations. Healthc. Technol. Lett..

[35] Sandercock G.R.H., Bromley P.D., Brodie D.A. (2005). The Reliability of Short-Term Measurements of Heart Rate Variability. Int. J. Cardiol..

[36] Protheroe C.L., Ravensbergen H.R.J.C., Inskip J.A., Claydon V.E. (2013). Tilt Testing with Combined Lower Body Negative Pressure: A “Gold Standard” for Measuring Orthostatic Tolerance. JoVE (J. Vis. Exp.).

[37] Malik M., Huikuri H., Lombardi F., Schmidt G. (2017). The Purpose of Heart Rate Variability Measurements. Clin. Auton. Res..

[38] Shaffer F., Meehan Z.M., Zerr C.L. (2020). A Critical Review of Ultra-Short-Term Heart Rate Variability Norms Research. Front. Neurosci..

[39] Volpes G., Barà C., Valenti S., Javorka M., Busacca A., Faes L., Pernice R. Feasibility of Ultra-Short Term Complexity Analysis of Heart Rate Variability in Resting State and During Orthostatic Stress. Proceedings of the 2022 12th Conference of the European Study Group on Cardiovascular Oscillations (ESGCO).

[40] Javorka M., Krohova J., Czippelova B., Turianikova Z., Lazarova Z., Javorka K., Faes L. (2017). Basic Cardiovascular Variability Signals: Mutual Directed Interactions Explored in the Information Domain. Physiol. Meas..

[41] Javorka M., Krohova J., Czippelova B., Turianikova Z., Lazarova Z., Wiszt R., Faes L. (2018). Towards Understanding the Complexity of Cardiovascular Oscillations: Insights from Information Theory. Comput. Biol. Med..

[42] Vollmer M. A Robust, Simple and Reliable Measure of Heart Rate Variability Using Relative RR Intervals. Proceedings of the 2015 Computing in Cardiology Conference (CinC).

[43] Valente M., Javorka M., Porta A., Bari V., Krohova J., Czippelova B., Turianikova Z., Nollo G., Faes L. (2018). Univariate and Multivariate Conditional Entropy Measures for the Characterization of Short-Term Cardiovascular Complexity under Physiological Stress. Physiol. Meas..

[44] Xiong W., Faes L., Ivanov P.C. (2017). Entropy Measures, Entropy Estimators, and Their Performance in Quantifying Complex Dynamics: Effects of Artifacts, Nonstationarity, and Long-Range Correlations. Phys. Rev. E.

[45] Azami H., Faes L., Escudero J., Humeau-Heurtier A., Silva L.E.V. (2020). Entropy Analysis of Univariate Biomedical Signals: Review and Comparison of Methods. Frontiers in Entropy across the Disciplines: Panorama of Entropy: Theory, Computation, and Applications.

[46] Hutcheson G.D. (2011). Ordinary Least-Squares Regression. The SAGE Dictionary of Quantitative Management Research.

[47] Esco M.R., Flatt A.A. (2014). Ultra-Short-Term Heart Rate Variability Indexes at Rest and Post-Exercise in Athletes: Evaluating the Agreement with Accepted Recommendations. J. Sports Sci. Med..

[48] Munoz M.L., van Roon A., Riese H., Thio C., Oostenbroek E., Westrik I., de Geus E.J.C., Gansevoort R., Lefrandt J., Nolte I.M. (2015). Validity of (Ultra-) Short Recordings for Heart Rate Variability Measurements. PLoS ONE.

[49] Benesty J., Chen J., Huang Y., Cohen I. (2009). Pearson Correlation Coefficient. Noise Reduction in Speech Processing.

[50] Shaffer F., Shearman S., Meehan Z., Gravett N., Urban H., Moss D., Shaffer F. (2019). The Promise of Ultra-Short-Term (UST) Heart Rate Variability Measurements: A Comparison of Pearson Product-Moment Correlation Coefficient and Limits of Agreement (LoA) Concurrent Validity Criteria. Physiological Recording Technology and Applications in Biofeedback and Neurofeedback.

[51] Fritz C.O., Morris P.E., Richler J.J. (2012). Effect Size Estimates: Current Use, Calculations, and Interpretation. J. Exp. Psychol. Gen..

[52] Cohen J., Hillsdale (1988). Statistical Power Analysis for the Behavioral Sciences.

[53] Baumert M., Czippelova B., Ganesan A., Schmidt M., Zaunseder S., Javorka M. (2014). Entropy Analysis of RR and QT Interval Variability during Orthostatic and Mental Stress in Healthy Subjects. Entropy.

[54] Wasmund W.L., Westerholm E.C., Watenpaugh D.E., Wasmund S.L., Smith M.L. (2002). Interactive Effects of Mental and Physical Stress on Cardiovascular Control. J. Appl. Physiol..

[55] Hjortskov N., Rissén D., Blangsted A.K., Fallentin N., Lundberg U., Søgaard K. (2004). The Effect of Mental Stress on Heart Rate Variability and Blood Pressure during Computer Work. Eur. J. Appl. Physiol..

[56] Paton J.F.R., Boscan P., Pickering A.E., Nalivaiko E. (2005). The Yin and Yang of Cardiac Autonomic Control: Vago-Sympathetic Interactions Revisited. Brain Res. Rev..

[57] Berntson G.G., Cacioppo J.T., Binkley P.F., Uchino B.N., Quigley K.S., Fieldstone A. (1994). Autonomic Cardiac Control. III. Psychological Stress and Cardiac Response in Autonomic Space as Revealed by Pharmacological Blockades. Psychophysiology.

[58] Faes L., Porta A., Cucino R., Cerutti S., Antolini R., Nollo G. (2004). Causal Transfer Function Analysis to Describe Closed Loop Interactions between Cardiovascular and Cardiorespiratory Variability Signals. Biol. Cybern..

[59] Porta A., Furlan R., Rimoldi O., Pagani M., Malliani A., Van De Borne P. (2002). Quantifying the Strength of the Linear Causal Coupling in Closed Loop Interacting Cardiovascular Variability Signals. Biol. Cybern..

[60] Porta A., Catai A.M., Takahashi A.C.M., Magagnin V., Bassani T., Tobaldini E., Van De Borne P., Montano N. (2011). Causal Relationships between Heart Period and Systolic Arterial Pressure during Graded Head-up Tilt. Am. J. Physiol. Integr. Comp. Physiol..

[61] Faes L., Nollo G., Porta A. (2011). Information Domain Approach to the Investigation of Cardio-Vascular, Cardio-Pulmonary, and Vasculo-Pulmonary Causal Couplings. Front. Physiol..

[62] Westerhof B.E., Gisolf J., Karemaker J.M., Wesseling K.H., Secher N.H., Van Lieshout J.J. (2006). Time Course Analysis of Baroreflex Sensitivity during Postural Stress. Am. J. Physiol. Circ. Physiol..

[63] Mijatovic G., Pernice R., Perinelli A., Antonacci Y., Busacca A., Javorka M., Ricci L., Faes L. (2022). Measuring the Rate of Information Exchange in Point-Process Data With Application to Cardiovascular Variability. Front. Netw. Physiol..

[64] Krohova J., Faes L., Czippelova B., Turianikova Z., Mazgutova N., Pernice R., Busacca A., Marinazzo D., Stramaglia S., Javorka M. (2019). Multiscale Information Decomposition Dissects Control Mechanisms of Heart Rate Variability at Rest and During Physiological Stress. Entropy.

[65] Pinto H., Pernice R., Eduarda Silva M., Javorka M., Faes L., Rocha A.P. (2022). Multiscale Partial Information Decomposition of Dynamic Processes with Short and Long-Range Correlations: Theory and Application to Cardiovascular Control. Physiol. Meas..

[66] Porta A., Gnecchi-Ruscone T., Tobaldini E., Guzzetti S., Furlan R., Montano N. (2007). Progressive Decrease of Heart Period Variability Entropy-Based Complexity during Graded Head-up Tilt. J. Appl. Physiol..

[67] Pernice R., Volpes G., Krohova J.C., Javorka M., Busacca A., Faes L. Feasibility of Linear Parametric Estimation of Dynamic Information Measures to Assess Physiological Stress from Short-Term Cardiovascular Variability. Proceedings of the 2021 43rd Annual International Conference of the IEEE Engineering in Medicine & Biology Society (EMBC).

[68] Fauvel J.P., Cerutti C., Quelin P., Laville M., Gustin M.P., Paultre C.Z., Ducher M. (2000). Mental Stress–Induced Increase in Blood Pressure Is Not Related to Baroreflex Sensitivity in Middle-Aged Healthy Men. Hypertension.

[69] Baselli G., Porta A., Pagani M. (2005). Coupling Arterial Windkessel with Peripheral Vasomotion: Modeling the Effects on Low-Frequency Oscillations. IEEE Trans. Biomed. Eng..

[70] Faes L., Kugiumtzis D., Nollo G., Jurysta F., Marinazzo D. (2015). Estimating the Decomposition of Predictive Information in Multivariate Systems. Phys. Rev. E.

[71] Clemson P.T., Hoag J.B., Cooke W.H., Eckberg D.L., Stefanovska A. (2022). Beyond the Baroreflex: A New Measure of Autonomic Regulation Based on the Time-Frequency Assessment of Variability, Phase Coherence and Couplings. Front. Netw. Physiol..

[72] Faes L., Gómez-Extremera M., Pernice R., Carpena P., Nollo G., Porta A., Bernaola-Galván P. (2019). Comparison of Methods for the Assessment of Nonlinearity in Short-Term Heart Rate Variability under Different Physiopathological States. Chaos Interdiscip. J. Nonlinear Sci..

[73] Bourdillon N., Schmitt L., Yazdani S., Vesin J.-M., Millet G.P. (2017). Minimal Window Duration for Accurate HRV Recording in Athletes. Front. Neurosci..

[74] Salahuddin L., Cho J., Jeong M.G., Kim D. Ultra Short Term Analysis of Heart Rate Variability for Monitoring Mental Stress in Mobile Settings. Proceedings of the 2007 29th Annual International Conference of the IEEE Engineering in Medicine and Biology Society.

[75] Baek H.J., Cho C.-H., Cho J., Woo J.-M. (2015). Reliability of Ultra-Short-Term Analysis as a Surrogate of Standard 5-Min Analysis of Heart Rate Variability. Telemed. e-Health.

[76] Goldberger J.J., Le F.K., Lahiri M., Kannankeril P.J., Ng J., Kadish A.H. (2006). Assessment of Parasympathetic Reactivation after Exercise. Am. J. Physiol. Circ. Physiol..

[77] McNames J., Aboy M. (2006). Reliability and Accuracy of Heart Rate Variability Metrics versus ECG Segment Duration. Med. Biol. Eng. Comput..

[78] Castaldo R., Montesinos L., Pecchia L. (2019). Ultra-Short Entropy for Mental Stress Detection. Proceedings of the World Congress on Medical Physics and Biomedical Engineering 2018.

[79] Lee S., Hwang H.B., Park S., Kim S., Ha J.H., Jang Y., Hwang S., Park H.-K., Lee J., Kim I.Y. (2022). Mental Stress Assessment Using Ultra Short Term HRV Analysis Based on Non-Linear Method. Biosensors.

[80] Faes L., Porta A., Javorka M., Nollo G. (2017). Efficient Computation of Multiscale Entropy over Short Biomedical Time Series Based on Linear State-Space Models. Complexity.

[81] Gil E., Orini M., Bailon R., Vergara J.M., Mainardi L., Laguna P. (2010). Photoplethysmography Pulse Rate Variability as a Surrogate Measurement of Heart Rate Variability during Non-Stationary Conditions. Physiol. Meas..

[82] Mejía-Mejía E., May J.M., Torres R., Kyriacou P.A. (2020). Pulse Rate Variability in Cardiovascular Health: A Review on Its Applications and Relationship with Heart Rate Variability. Physiol. Meas..

[83] Jiao Y., Wang X., Liu C., Du G., Zhao L., Dong H., Zhao S., Liu Y. (2023). Feasibility Study for Detection of Mental Stress and Depression Using Pulse Rate Variability Metrics via Various Durations. Biomed. Signal Process. Control.

[84] Wang J.H.-S., Yeh M.-H., Chao P.C.-P., Tu T.-Y., Kao Y.-H., Pandey R. (2020). A Fast Digital Chip Implementing a Real-Time Noise-Resistant Algorithm for Estimating Blood Pressure Using a Non-Invasive, Cuffless PPG Sensor. Microsyst. Technol..

